# Osteoprotective Activity of *Sambucus javanica* Reinw Ex Blume subsp. *javanica* Leaf Extracts by Suppressing ROS Production

**DOI:** 10.3390/antiox14030252

**Published:** 2025-02-21

**Authors:** Treethip Sukkho, Chartchai Khanongnuch, Saisamorn Lumyong, Jetsada Ruangsuriya, Sutasinee Apichai, Young-Joon Surh, Thanawat Pattananandecha, Chalermpong Saenjum

**Affiliations:** 1 Department of Biotechnology, Multidisciplinary and Interdisciplinary School, Chiang Mai University, Chiang Mai 50200, Thailand; treethip.sk@gmail.com; 2 Research Center for Innovation in Analytical Science and Technology for Biodiversity-Based Economic and Society (I-ANALY-S-T_B.BES-CMU), Multidisciplinary Research Institute (MDRI), Chiang Mai University, Chiang Mai 50200, Thailand; ck_biot@yahoo.com (C.K.); jetsada.ruang@cmu.ac.th (J.R.); sutasinee.apichai@gmail.com (S.A.); 3 Research Center for Multidisciplinary Approaches to Miang, Multidisciplinary Research Institute (MDRI), Chiang Mai University, Chiang Mai 50200, Thailand; 4 Department of Biology, Faculty of Sciences, Chiang Mai University, Chiang Mai 50200, Thailand; scboi009@gmail.com; 5 Research Center of Microbial Diversity and Sustainable Utilization, Faculty of Science, Chiang Mai University, Chiang Mai 50200, Thailand; 6 Department of Biochemistry, Faculty of Medicine, Chiang Mai University, Chiang Mai 50200, Thailand; 7 Office of Research Administration, Chiang Mai University, Chiang Mai 50200, Thailand; 8 College of Pharmacy, Seoul National University, Seoul 08828, Republic of Korea; surh@snu.ac.kr; 9 Department of Pharmaceutical Sciences, Faculty of Pharmacy, Chiang Mai University, Chiang Mai 50200, Thailand

**Keywords:** *Sambucus javanica* subsp. *javanica*, osteoblastogenesis, bone formation, reactive oxygen species (ROS), natural active pharmaceutical ingredient (NAPI)

## Abstract

*Sambucus javanica* subsp. *javanica* (SJ) has been used in traditional medicine in the northern region of Thailand for healing bone fractures; however, studies on how this plant stimulates bone formation are still scarce. The present study aimed to investigate the potential of crude extracts and fractions obtained from SJ leaves for osteoporotic protection. All samples were investigated in murine preosteoblast MC3T3-E1 cells for bone formation and resorption biomarkers, namely alkaline phosphatase (ALP), osteocalcin (OC), osteoprotegerin (OPG), receptor activator of nuclear factor-κB ligand (RANKL), and the OPG/RANKL ratio. Additionally, calcium deposits were determined using the alizarin red S staining technique. The results indicated that the crude water and the crude ethanol extracts contained gallic acid, rutin, and chlorogenic acid as major compounds. The extracts stimulated osteoblastic cell differentiation and enhanced osteoprotective activity, as measured by a significant increase in ALP activity, OC, OPG, the OPG/RANKL ratio, and the degree of calcification. Additionally, they exhibited a negative impact on bone resorption by significantly reducing RANKL and reactive oxygen species (ROS) production. Therefore, our findings add novel evidence indicating that the SJ crude extracts from water and ethanol extraction could be further utilized as a natural active pharmaceutical ingredient (NAPI) for the development of bone health products.

## 1. Introduction

Osteoporosis is a result of an imbalance between bone formation and resorption and is associated with advancing age, especially in females. An imbalance of bone resorption and formation processes leads to a gradual decline in bone mass noticeable after 45 years of age. Furthermore, the rate of bone resorption significantly rises after menopause due to estrogen insufficiency, leading to a porous bone structure and low bone density, which accounts for increased fragility and a higher risk of fractures [1]. High incidences of fractures typically occur in the areas of the wrist, spine, and hips. Hip fractures are considered to have serious impacts, affecting patient disability and morbidity. Apart from the effect of hormonal changes, osteoporosis can result from malnutrition, especially a lack of calcium and vitamin D, chronic inflammation, and the long-term use of certain drugs, such as steroids [2].

Reactive oxygen species (ROS) are vital for regulating bone cell differentiation, self-renewal, and proliferation. An imbalance of ROS production significantly contributes to osteoporosis. Heavy production of ROS triggers osteoblast and osteocyte apoptosis. Additionally, ROS inhibits mineralization and osteogenesis, resulting in the promotion of bone loss. Interestingly, antioxidants such as γ-glutamyl-cysteinyl-glycine (GSH), vitamin C, and vitamin A facilitate the activation of osteoblastic differentiation and mineralization and prevent osteoclastogenesis [3]. Hydrogen peroxide (H_2_O_2_) plays a central role in bone loss linked to estrogen deficiency, a major cause of osteoporosis and fracture risk. Excessive ROS cause oxidative stress, inhibiting osteoblast differentiation, promoting apoptosis in osteoblasts and osteocytes, increasing osteoclast formation, and inhibiting mineralization [4].

Numerous pieces of evidence have revealed that several plants and their constituents exhibit anti-osteoporosis properties. For instance, isoflavones from soybeans act as phytoestrogens, enhancing osteoblast activity and reducing osteoclast-mediated bone resorption [5]. Similarly, kaempferol, a flavonoid found in broccoli, spinach, and tea, exhibits antioxidant and anti-inflammatory effects, protecting bone cells from ROS and promoting bone formation. One possible mechanism on those active ingredients is antioxidant properties to combat oxidative stress in order to increase bone mineral density and decrease the risk of fragility-related fractures [6].

*Sambucus javanica*, a shrub species of elderberry in the Viburnaceae family, is traditionally used in northern Thailand for treating bone fractures. In Mae Kampong, villagers have crushed the leaves for application to the skin where fractures, bruises, and muscle pain occur. Traditional healers in northern Thailand commonly use local herbal plants to treat bone fractures, by crushing parts of medicinal plants and applying them as a poultice on the skin over the fractured area [7]. This practice is consistent with many reports from around the world. For example, *S. williamsii* is widely used in traditional Chinese medicine for treating fractures and enhancing bone healing. Poultices made from its stem are traditionally applied to fractured areas [8]. Additionally, in Turkish folk medicine, *S. ebulus* is used for bone and joint disorders, with poultices from its leaves and stems applied externally to reduce inflammation and promote fracture healing [9]. This method helps deliver active compounds directly to the injury site. The warmth and moisture enhance absorption. Poultices typically provide healing effects, such as anti-inflammatory and analgesic properties, help to reduce pain and swelling, support an optimal environment for bone repair with regenerative properties, and overall speed up recovery [10].

Biomarkers play a critical role in evaluating bone metabolism and remodeling processes. Alkaline phosphatase (ALP) is an early osteoblast differentiation marker essential for mineralization [11]. Osteocalcin (OC), produced by mature osteoblasts, is key in matrix mineralization and calcium ion balance [12]. Receptor activator of nuclear factor-κB ligand (RANKL), secreted by osteoblasts, binds to its transmembrane receptor RANK on osteoclast precursors to stimulate their differentiation and promote bone resorption. Osteoprotegerin (OPG), a decoy receptor for RANKL produced by osteoblasts, prevents RANKL–RANK binding to inhibit osteoclast differentiation and maturation [13]. The OPG/RANKL ratio is a crucial factor for controlling osteoclast differentiation and bone remodeling, where a higher ratio supports bone formation, while a lower ratio increases bone resorption [14].

Our previous study surveyed medicinal plants traditionally used by healers in northern Thailand for the treatment of bone and muscle disorders. Among these, *Sambucus javanica* Reinw ex Blume subsp. *javanica* (SJ) demonstrated significant potential to promote bone formation. The study found that SJ crude extracts significantly increased ALP activity in MG-63 osteoblast cells [7]. These findings highlight the potential value of fractionating SJ crude extracts to identify active ingredients for further development. Thus, this study aimed to investigate the effects of SJ crude extracts and fractions on osteoporotic protection in the murine preosteoblast MC3T3-E1 cells in the context of their capability to inhibit ROS production. A number of key bone formation and resorption biomarkers, including ALP and OC, along with OPG and RANKL, were used for early osteoporosis evaluation.

## 2. Materials and Methods

### 2.1. Chemicals and Biological Reagents

Alpha minimum essential medium (αMEM), fetal bovine serum (FBS), 0.5% trypsin-EDTA solution, penicillin–streptomycin solution, phosphate-buffered saline (pH 7.4), hydrochloric acid, aluminum chloride (AlCl_3_), sodium hydroxide, dimethylsulfoxide, ethanol, 4-nitrophenol magnesium chloride hexahydrate, bovine serum albumin (BSA), CelLytic™ M cell lysis reagent, diethanolamine, Bradford reagent, L-ascorbic acid (AsA), β-glycerophosphate disodium salt hydrate (β-GP), dexamethasone (DEX), alizarin red S, ammonium hydroxide solution, gallic acid monohydrate, 2′,7′-dichlorodihydrofluorescein diacetate (DCFH-DA), *p*-nitrophenyl phosphate disodium salt hexahydrate, PrestoBlue^TM^ cell viability reagent, chlorogenic acid, rutin hydrate, quercetin hydrate, naringenin hydrate, hesperidin, kaempferol hydrate, caffeic acid, pyrogallol, and isoquercitrin were purchased from the manufacturers. All information about the chemical and biological reagents is listed in Table S1.

### 2.2. Preparation of SJ Extracts and Fractions

SJ leaves were collected from Mae Kampong village, Mae On District, Chiang Mai, Thailand, in January 2020 (latitude: 18.86648, longitude: 99.35662). A voucher specimen (No. 0023270) was deposited in the Herbarium of the Faculty of Pharmacy, Chiang Mai University, Thailand. The SJ leaves were cleaned, dried, and extracted with either deionized water at 100 °C or 80% ethanol at 80 °C. The extracts were filtered, evaporated under reduced pressure, and dried under a vacuum to acquire a crude extract. Before fractionation, the dried crude water extract was reconstituted in deionized water and subsequently fractionated using hexane, ethyl acetate, and butanol. Meanwhile, the dried crude ethanolic extract was reconstituted in 80% ethanol and partitioned in sequence with hexane, ethyl acetate, and dichloromethane. The obtained solutions were filtered, evaporated, dried, and stored at −20 °C for further use.

### 2.3. Cell Culture

The mouse MC3T3-E1 subclone 4 (ATCC^®^ CRL-2593™; Manassas, VA, USA) was cultured according to the manufacturer’s protocol in αMEM supplemented with 10% FBS and 1% penicillin–streptomycin at 37 °C and 5% CO_2_ (New Brunswick Galaxy 48 S CO_2_ Incubators, Oldenburg, Germany). When reaching 70–80% confluence, the cells were trypsinized for subculturing and/or experiments.

### 2.4. Measurement of Cell Viability

Cells (5.0 × 10^3^ cells/well) were seeded into each well of a 24-well plate and incubated for 24 h at 37 °C with 5% CO_2_. Then, the culture medium was replaced with the SJ extracts or the fractions at different concentrations (25–200 µg/mL) for 72 h. When due, the media were removed, and PrestoBlue^TM^ solution was added into each well and incubated for 2 h. The absorbance was measured at 560 and 595 nm with a microplate reader (Spectramax M3, San Jose, CA, USA), and the cell viability was calculated [7].

### 2.5. Determination of Total Protein and ALP Activity

Both the protein and the ALP activity were determined in a cell lysate prepared by the treatment of cell layers with commercial CelLytic™ M, the cell lysis reagent. The protein levels were analyzed with Bradford reagent. BSA was used as a positive control for calculation. Briefly, the cell lysates were mixed with working Bradford reagent and incubated for 5 min at room temperature. The absorbance was measured at 595 nm, and the protein amount was calculated using the BSA standard curve [15].

ALP activity in the cell lysates was determined by the rate of *p*-nitrophenyl phosphate (pNPP) conversion. The lysates were incubated with pNPP in diethanolamine buffer (pH = 9.8) for 120 min at 37 °C. The reaction was stopped by adding 1 M NaOH, and the absorbance was measured at 405 nm. The ALP activity was calculated using a *p*-nitrophenol (pNP) standard curve, the reaction incubation time, and the total protein content [7].

### 2.6. Measurements of Osteocalcin (OC), Osteoprotegerin (OPG), and Receptor Activator of Nuclear Factor Kappa-Β Ligand (RANKL) by ELISA

OC, OPG, and RANKL, the classical biomarkers associated with bone formation, were analyzed in the cell culture supernatants using SimpleStep ELISA^®^ kits by following the manufacturer’s protocols (Abcam Ltd., Cambridge, UK). The ELISA kits used were mouse osteocalcin (ab285236), mouse osteoprotegerin (ab203365), and mouse RANKL (ab269553).

### 2.7. Alizarin Red S Staining and Calcium Assay

Alizarin red S staining was used to visualize the degree of calcification. MC3T3-E1 cells (1.0 × 10^4^ cells/well) were treated with osteogenic medium (OSM), comprising the completed αMEM, 0.2 mM AsA, 10 mM β-GP, and 0.01 μM DEX for 24 h. Then, the medium supernatant was replaced with the completed medium containing tested samples at the collected concentrations, and the cells were incubated with 5% CO_2_ at 37 °C for 7 days. The cells were fixed with 50% ethanol at 4 °C and stained with 1% alizarin red S solution in 1% ammonium hydroxide for 30–45 min to visualize calcium accumulation. The unstained dye was washed with deionized water, the stained layer was completely dried at room temperature, and the calcified region was imaged under a microscope. Finally, the stained dye was extracted using 10% acetic acid in deionized water with methanol for 30–45 min, and the absorbance was measured at 450 nm [16].

### 2.8. Analysis and Identification of Phytochemicals in SJ Extracts

The phenolic and flavonoid compounds in the extracts were analyzed by reverse-phase high-performance liquid chromatography (RP-HPLC) equipped with a multi-wavelength detector, an Agilent model 1200 (Agilent Technologies, Santa Clara, CA, USA), and the major constituents were identified by liquid chromatography–mass spectrometry–quadrupole-time of flight (LC-MS/QTOF) using an Agilent 6546 mass spectrometer (Agilent Technologies, CA, USA) [17]. A Symmetry C18 column was employed, and the mobile phase consisted of 0.1% acetic acid in a 70:30 mixture of deionized water and acetonitrile, with a flow rate of 1.0 mL/min. Detection occurred at 278 nm for gallic acid and pyrogallol, 325 nm for caffeic acid, rosmarinic acid, and chlorogenic acid, and 350 nm for rutin, luteolin, quercetin, isoquercitrin, apigenin, and kaempferol. The compounds were separated and detected as chromatogram peaks, which were analyzed for qualitative identification and quantified by the area under the peak [18].

### 2.9. Measurement of Intracellular ROS Production

The inhibitory effect of the tested samples on the intracellular ROS production was determined using the DCFH-DA method. MC3T3-E1 cells (1 × 10^6^ cells/mL) were seeded in 96-well plates and pretreated with SJ extracts or fractions at different concentrations (2.5–25 µg/mL) for 12 h. Then, the treatment with 2 mM AlCl_3_ for 24 h was introduced to induce ROS production. Next, 40 µM of DCFH-DA solution was added, and the cells were allowed to react with the solution for 30 min at 37 °C and 5% CO_2_. The green fluorescence intensity was measured at 480 nm excitation and 525 nm emission. Additionally, 5 mM N-acetylcysteine (NAC), 10 µg/mL rutin (Rut), and 5 µg/mL L-ascorbic acid (AsA) were used as positive controls [19,20].

### 2.10. Statistical Analysis

The data are presented as the mean ± SD from three independent experiments. One-way analysis of variance (ANOVA) followed by a Tukey post hoc test was performed with SPSS Statistics software, version 27.0 (The International Business Machines Corporation (IBM), New Orchard Road, Armonk, NY, USA), for determining significant differences among groups when *p* < 0.05, 0.01, 0.001, and 0.0001.

## 3. Results

### 3.1. Yield of Plant Crude Extracts and Fractions

After extracting SJ leaves with deionized water and 80% ethanol, each crude extract was partitioned using solvents with different polarities. The percentage yield of two different crude extracts and fractions of SJ leaves is presented in Table 1. Among the fractions of the crude water extract, the deionized water fraction (W-FW) yielded the highest percentage, with a value of 81.44%, whereas the lowest percentage yield was the hexane fraction (W-FH). In the fractions of the crude ethanolic extract, the ethanolic fraction (E-FE) yielded the highest percentage, with a value of 74.13%, whereas the lowest percentage yield was the hexane fraction (E-FH).

### 3.2. Expression of Bone Formation Biomarkers and Regulators of Bone Turnover

#### 3.2.1. ALP Activity

Prior to determining the effects of SJ extracts and fractions on bone formation, an MC3T3-E1 cell viability test was carried out using a PrestoBlue^TM^ assay to ensure the non-toxic dose of the extracts and fractions. It was found that the W-C did not affect the cell viability at all tested concentrations, ranging from 25 to 200 μg/mL. Only at a high concentration of 200 μg/mL did E-C exhibit cytotoxicity. Unlike the SJ fractions, it was found that all the fractions at concentrations of more than 50 μg/mL exerted cytotoxicity (Supplementary Figure S2). According to the cytotoxicity results, all the tested samples with a concentration of 25 μg/mL were selected to investigate their osteoporotic protection in MC3T3-E1 osteoblast cells.

**Figure 1:**
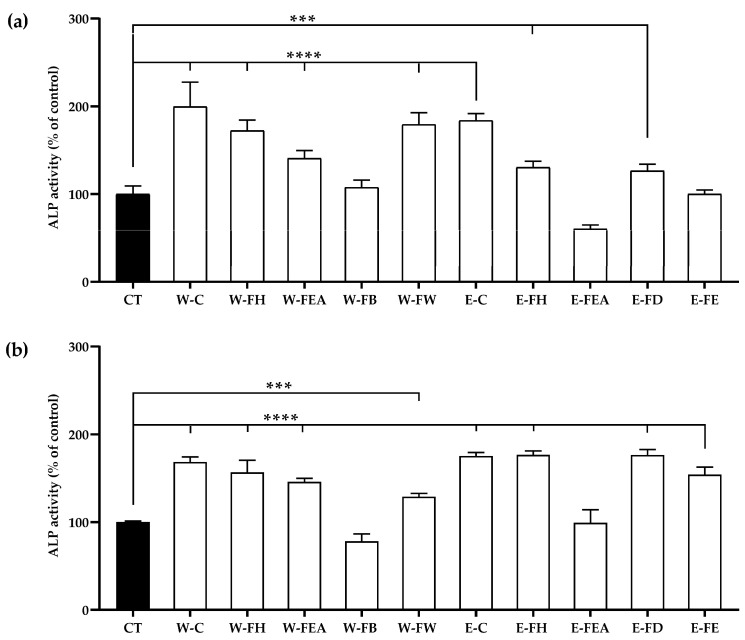
The ALP activity normalized with total protein contents of MC3T3-E1 cells, incubated for (**a**) 4 days or (**b**) 7 days in αMEM medium (CT) or in αMEM medium supplemented with 25 μg/mL of SJ extracts or fractions. Data are expressed as mean ± SD (*n* = 6). Significance levels at *** *p* < 0.001 and **** *p* < 0.0001 when compared to the control.

Both the SJ crude extracts and nearly all fractions significantly promoted ALP activity, except W-FB, E-FEA, and E-FE on day 4, as well as W-FB and E-FEA on day 7. On day 4, the ALP activity of MC3T3-E1 cells treated with W-C was significantly higher than that of the control group, followed by the treatment with E-C. The ALP activity of MC3T3-E1 cells treated with E-C on day 7 was the highest by a significant margin, followed by the treatments with W-C, E-FH, and E-FD (Figure 1a,b).

#### 3.2.2. Osteocalcin (OC) Levels

**Figure 2:**
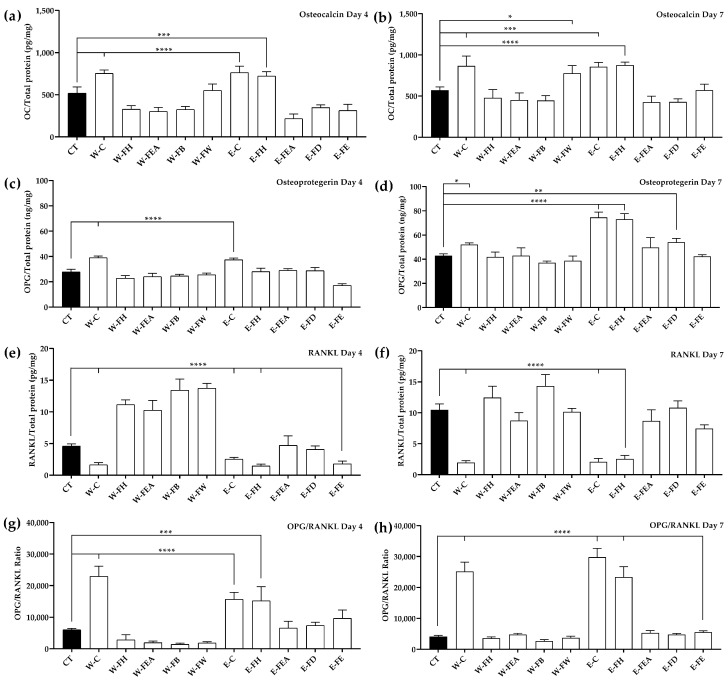
The levels of bone marker normalized with protein contents in MC3T3-E1 cells. (**a**,**b**) Osteocalcin (OC), (**c**,**d**) osteoprotegerin (OPG), (**e**,**f**) receptor activator of nuclear factor kappa-Β ligand (RANKL), and (**g**,**h**) OPG/RANKL ratio, observed at 4 and 7 days in αMEM (CT) or in αMEM supplemented with 25 μg/mL of SJ extracts or fractions. Data are expressed as mean ± SD where *n* = 4. Significance levels at * *p* < 0.05; ** *p* < 0.01; *** *p* < 0.001; and **** *p* < 0.0001 when compared to the control.

It was found that W-C and E-C activated MC3T3-E1 cells to produce OC at significantly higher levels when compared to the control at both time points (Figure 2a,b). In addition, E-FH also increased OC at similar levels to that of W-C and E-C on day 4. Likewise, W-FW and E-FH also increased OC at similar levels to that of E-C on day 7.

#### 3.2.3. Osteoprotegerin (OPG) Levels

Like the OC levels, W-C and E-C activated MC3T3-E1 cells to produce OPG at significantly higher levels than the control at both time points (Figure 2c,d). E-FH increased OPG at a similar level to that of E-C at day 7. In addition, E-FD significantly increased (*p* < 0.01) OPG levels when compared to the control at day 7.

#### 3.2.4. Receptor Activator of Nuclear Factor Kappa-Β Ligand (RANKL) and the OPG/RANKL Ratio

In contrast to the OC and OPG levels, W-C and E-C significantly reduced (*p* < 0.0001) the RANKL levels when compared to the control at both time points (Figure 2e,f). E-FH also significantly reduced (*p* < 0.0001) the RANKL levels to a similar degree to that of W-C and E-C at day 4. At day 7, E-FE also reduced the RANKL levels significantly (*p* < 0.01) at day 7.

Conversely, the OPG/RANKL ratio of MC3T3-E1 represents the ability of cells to promote or suppress osteoclastogenesis. A high ratio tends to suppress osteoclastogenesis. The ratios of OPG/RANKL in W-C and E-C were significantly higher (*p* < 0.0001) than that of the control at both time points (Figure 2g,h). Additionally, E-FH significantly increased the OPG/RANKL levels when compared to the control at day 4 (*p* < 0.001) and day 7 (*p* < 0.0001).

#### 3.2.5. Calcification in MC3T3-E1 Cells by Alizarin Red S Staining

**Figure 3:**
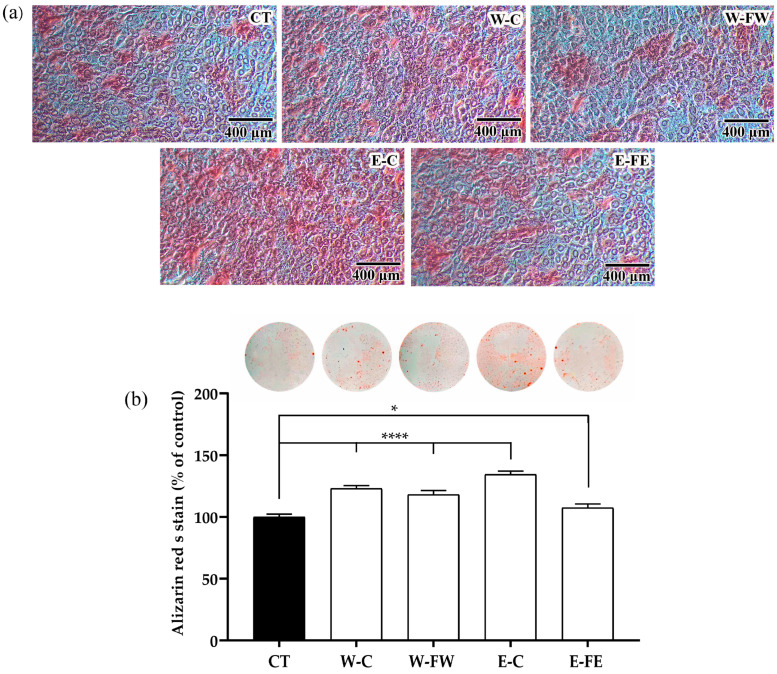
Calcification of MC3T3-E1 cells after a 7-day culture in osteogenic medium. (**a**) Representative pictures with the 400 µm scale bar from light microscopy at 20× magnification of alizarin red S-stained cell layers (**b**) Alizarin red S staining in 24-well plates and the percentage of stained dye intensity after solubilization in MC3T3-E1 cells cultured with osteogenic medium (CT) or in osteogenic medium supplemented with 25 μg/mL of SJ extracts or fractions. Data are expressed as mean ± SD where *n* = 3. Significance levels at * *p* < 0.05 and **** *p* < 0.0001 when compared to the control.

Calcification represents the final phase of bone synthesis, leading to the development of calcified nodules that enhance the structural integrity of the bone. It was found that W-C, W-FW, and E-C promoted the staining of the cell with alizarin red S, indicating the calcification area (Figure 3a). Likewise, the intensity of the stained dye was significantly higher (*p* < 0.0001) in W-C-, W-FW-, and E-C-treated cells (Figure 3b). Similarly, the intensity of the stained dye in the cells treated with E-FE was also significantly higher (*p* < 0.05) compared to the control.

### 3.3. Inhibition of Intracellular ROS Production by Crude Extracts and Fractions

**Figure 4:**
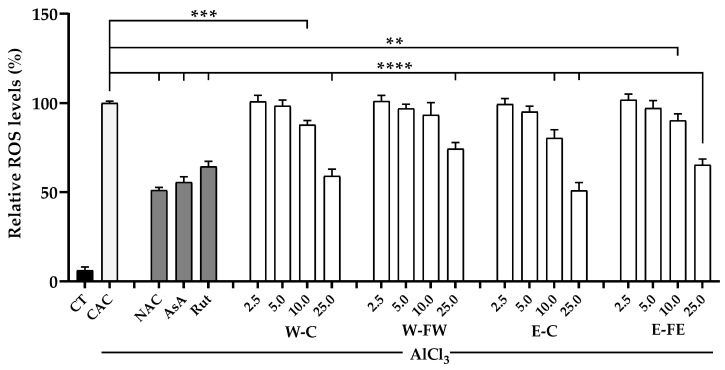
Relative levels of intracellular ROS production in MC3T3-E1 cells. The cells were pretreated with 2.5–25 μg/mL of the W-C, E-C, W-FW, and E-FE samples for 12 h prior to ROS induction by 2 mM AlCl_3_ for 24 h. The cells without pretreatment, treated only with AlCl_3_ were used as a control (CAC), while the non-treated cells (CTs) were also observed for their ROS production without AlCl_3_ induction. Additionally, 5 mM N-acetylcysteine (NAC), 10 µg/mL rutin (Rut), and 5 µg/mL L-ascorbic acid (AsA) were used as positive controls. Data are expressed as mean ± SD, where *n* = 3. Significance levels at ** *p* < 0.01; *** *p* < 0.001; and **** *p* < 0.0001 when compared to the control.

The selected tested samples, namely SJ W-C, E-C, W-FW, and E-FE, in a concentration of 25 μg/mL, were significantly capable (*p* < 0.05) of reducing the intracellular ROS production induced by AlCl_3_ in MC3T3-E1 cells with high potential to be standard positive controls. In addition, 10 μg/mL of the W-C and E-C samples was also significantly capable of reducing intracellular ROS production when compared to the cells without pretreatment but treated only with AlCl_3_ (Figure 4).

### 3.4. Analysis of Bioactive Components by HPLC

The phenolic and flavonoid contents in the crude extracts and fractions were determined by HPLC analysis and identified using a mass spectrometer (Figure S3). The quantification of phenolic and flavonoid contents in the leaf crude extracts and fractions was based on the peak area in comparison with standard compounds. The HPLC analysis showed that the W-C, W-FW, E-C, and E-FE were rich in gallic acid, rutin, and chlorogenic acid (Table 2). In contrast, the E-FH contained distinct major components, namely isoquercitrin and kaempferol.

## 4. Discussion

Osteoporosis remains a major global health concern, affecting millions of individuals and increasing the risk of fractures and mobility issues. Thus, this study aimed to evaluate the potential osteoprotective effects of SJ crude extract and fractions on MC3T3-E1 cells by analyzing biomarkers related to the bone formation and resorption process, including ALP activity, calcium deposit, OC, OPG, and RANKL, as well as their capability to inhibit ROS production. Our findings demonstrated that the crude extract effectively enhanced osteoblast activity while reducing ROS production, suggesting its potential application in bone health management. Similar to other species that have been reported in bone remodeling management, including *S. williamsii* Hance and *S. formosana* Nakai, SJ also showed the promotion of osteoblast differentiation and mineralization in MC3T3-E1 osteoblastic cells. However, the type of extractant (deionized water and 80% ethanol) affected the composition and potency of the extract compounds [3,21]. DI water is a polar solvent that primarily extracts hydrophilic compounds, such as flavonoids and phenolic acids. In contrast, 80% ethanol is less polar, and it extracts a wider range of compounds, including both hydrophilic and semi-lipophilic compounds, including polyphenols and flavonoids [22]. The SJ extracts, containing gallic acid, rutin, and chlorogenic acid as major compounds, promoted osteoblast differentiation and increased bone formation biomarkers, including ALP activity, OC, OPG, and the OPG/RANKL ratio, while reducing the RANKL levels. Additionally, the extracts contained minor compounds, namely caffeic acid, pyrogallol, quercetin, isoquercitrin, and kaempferol, which have been shown to support bone formation and inhibit bone resorption [23,24,25]. The current study showed that the SJ crude extract and fractions at 25 µg/mL, after 72 h of co-culturing in MC3T3-E1 cells, were at the optimal concentration for studying osteogenic activity, as they were non-toxic. This is consistent with previous research that reported the presence of several active compounds in plants of the same genus, such as *S. williamsii*, which increased cell viability, suggesting that these active compounds may promote MC3T3-E1 cell differentiation and proliferation via the BMP-2/Smad/p38/JNK/Runx2 signaling pathway [26]. By assessing the ALP activity in MC3T3-E1 cells, our findings indicated that the SJ crude extracts, specifically W-C and W-E, demonstrated the highest ability to stimulate ALP activity compared to other fractions, except for E-FEA, which showed no effect on ALP activity. An increase in ALP activity indicates a subsequent enhancement in osteoblastic activity and bone formation. Additionally, it was found that the ALP activity increased with the incubation time, with the ALP activity after 7 days of treatment being higher than that observed after 4 days. This is consistent with the report by Kwon et al., which stated that the ALP activities of MC3T3-E1 cells gradually increased over the incubation period until 14 days. After 21 days, the activity either decreased or stabilized [27]. Key components in the SJ crude extracts, such as gallic acid and chlorogenic acid, have been previously studied. Evidence suggests that gallic acid enhances ALP activity and promotes osteoblast differentiation. Its effects are associated with the G-protein-coupled receptor 35 (GPR35)/GSK3β/β-catenin signaling pathway [28]. Furthermore, based on the findings of Guan et al., it was discovered that plant extracts containing chlorogenic acid and isoquercitrin increase ALP activity, calcium deposition, and the ability of MC3T3-E1 cells [29]. Our findings indicate that W-C, W-E, and E-FH exhibited the highest OC levels. This is likely due to the active compounds like gallic acid and chlorogenic acid in the W-C and W-E. These compounds are known to promote osteogenic differentiation, which aligns with the findings of Yao, who demonstrated that gallic acid enhances the expression of osteogenic genes, including the OC gene, and promotes osteogenic differentiation by modulating autophagy via the JNK/mTOR signaling pathways [30]. Additionally, the study by Shen et al. reported that chlorogenic acid promoted osteoblast differentiation by activating the Wnt/BMP signaling pathway, leading to the increased expression of osteogenic markers, including ALP, OC, Runx2, and BMP2 in primary osteoblasts [31], while E-FH specifically contained isoquercitrin. Wang et al. reported that isoquercitrin upregulates the expression of the gene responsible for OC, which promotes the formation of bone minerals [32]. The ability of W-C, E-C, and E-FH to enhance OPG levels in MC3T3-E1 cells can be explained by the bioactive compounds present in each extract and fraction. The W-C and E-C contain significant amounts of gallic acid and chlorogenic acid. It has been found that chlorogenic acid may play a role in bone remodeling by directly influencing osteoblasts and regulating the OPG system [31]. The E-FH includes important components like isoquercitrin, which enhances the expression of genes linked to osteoblastic differentiation, along with ALP and OPG, contributing to the differentiation and maturation of osteoblasts [33]. Moreover, W-C, E-C, and E-FH also reduce the RANKL levels in MC3T3-E1cells. The key compounds found in W-C and E-C, such as gallic acid, help inhibit the RANKL-induced osteoclast formation and suppress osteoclastogenesis by blocking the Akt, ERK, and JNK signaling pathways, which is consistent with the findings reported by Zhang et al. [34]. Furthermore, chlorogenic acid also inhibits the activation of RANKL-induced NF-κB, a pathway associated with osteoclast differentiation and bone resorption [35]. Since these compounds reduce the RANKL levels and increase OPG levels, this results in an increase in the OPG/RANKL ratio. This is a crucial factor for regulating osteoclast differentiation and bone remodeling, as a higher ratio supports bone formation [36]. After evaluating the biomarkers related to bone formation, crude extracts and fractions with notable effects on promoting bone cell formation, including W-C, W-FW, E-C, and E-FE, were selected and stained with alizarin red S on day 7 of incubation to visualize calcium deposition, which helps confirm that these crude extracts and fractions influence osteoblastic differentiation via calcium deposits. According to Fu et al., mineral deposition serves as a late-stage indicator of osteogenic differentiation, confirming that MC3T3-E1 cells have transitioned into the mineralization phase [37]. It was observed that both the crude water and ethanolic extracts of SJ significantly enhanced osteoblast differentiation more than the other fractions. Although the hexane fraction also enhanced bone formation, it had a low percentage yield. The higher potency of crude extracts compared to fractions may result from synergistic effects among multiple bioactive compounds. It has been reported that traditional medicinal plant extracts has shown that they contain complex mixtures of bioactives targeting multiple sites, with overall activity driven by the combined action of these compounds; synergistic interactions are essential to their therapeutic efficacy [38]. This is consistent with results found in other studies. Researchers found the crude extracts of *S. williamsii* Hance to be superior in enhancing bone formation compared to isolated fractions. A crude ethanolic extract improved bone density and strength in animal models, likely due to synergistic interactions among bioactive compounds like lignans, flavonoids, and polysaccharides, which were often lost during fractionation [39]. ROS induce apoptosis in osteoblasts and disturb the equilibrium between osteoblasts and osteoclasts, leading to increased bone resorption and the development of conditions like osteoporosis. Our findings suggest that gallic acid and chlorogenic acid, the major active compounds present in W-C and E-C, are capable of decreasing ROS production. These compounds showed higher antioxidant activity and effectively protected MC3T3 cells from oxidative stress by inhibiting ROS generation and preventing ROS-induced apoptosis [40]. Similarly, isoquercitrin, a major active compound found in E-FH, also exhibited higher ROS-scavenging activity and may protect MC3T3-E1 cells from oxidative stress by inhibiting excessive intracellular ROS, thereby promoting bone development [41]. Similarly, apigenin also exhibits antioxidative and bone-regulating properties. In MC3T3-E1 cells, apigenin (1–10 µM) inhibits cell proliferation and osteoblast differentiation markers, including collagen production, ALP activity, and calcium deposition. Conversely, in human osteoblasts (hOBs), apigenin (5 µM) enhances cell proliferation, upregulates ALP and collagen 1 (COL1) gene expression, and promotes mineralization. Apigenin (10 µM) also suppresses multinucleated osteoclast formation in mouse splenic cells, and in OVX mice, its administration (10 mg/kg) effectively prevents trabecular bone loss. These findings highlight apigenin’s potential as a therapeutic agent for osteoporosis and bone regeneration [42,43].

It can be seen that both the major and minor active compounds of SJ are mostly phenolic compounds, which include phenolic acids and flavonoids. Plant-based therapies, particularly phenolic compounds, are considered safe for osteoporosis treatment due to minimal side effects. These compounds are abundant in natural sources, affordable, and sustainable. A study by Murakami et al. demonstrated that phenolic compounds benefit bone metabolism, preserve bone integrity, and reduce bone loss by lowering ROS and oxidative stress, reducing inflammation, enhancing osteoblast formation, and inhibiting osteoclast formation [44]. The biochemical activity of these compounds is largely attributed to the phenolic hydroxyl group, which allows phenols to bind to other molecules through covalent and non-covalent interactions. This group reduces levels of ROS, effectively suppressing intracellular oxidative stress [45]. This study highlights the value of indigenous knowledge in using medicinal plants for disease treatment. Scientific validation confirms that SJ protects bones by promoting biomarkers and regulators for bone formation, inhibiting those related to bone resorption, and reducing ROS production. These properties are consistent with the traditional use of this medicinal plant for treating bone fractures in local communities. These findings support the further exploration of gene expression related to bone modeling processes and associated pathways. It would be beneficial to utilize the crude extracts derived from SJ to prepare and develop natural active pharmaceutical ingredients (NAPIs) to prevent osteoporosis.

## 5. Conclusions

The current study aimed to investigate the effects of SJ crude extracts and fractions on osteoporotic protection in the murine preosteoblast MC3T3-E1. The results demonstrated that SJ leaf crude water and ethanolic extracts were rich in gallic acid, rutin, and chlorogenic acid. Some minor compounds, such as caffeic acid, naringenin, quercetin, hesperidin, and kaempferol, were also found. The extracts and some fractions showed osteoprotective effects, enhancing bone formation biomarkers, namely ALP, OC, OPG, and the OPG/RANKL ratio, and reducing the RANKL and ROS levels. Thus, SJ extracts could promote the development of natural active pharmaceutical ingredients (NAPIs) for osteoporosis prevention and promoting bone health. Aside from our experiments, the biological and pharmacological properties of SJ extract have been intensively investigated using cell-based study models. Future research should be more focused on in vivo and clinical studies. Their beneficial applications need to be supported by such evidence.

## Figures and Tables

**Table 1:** The percentage yield of crude extracts and fractions from SJ leaves.

Crude Extract	Percentage Yield (%*w/w*)	Fractions	Percentage Yield (%*w/w*)
Crude water extract (W-C)	11.52	Hexane (W-FH)	2.58
		Ethyl acetate (W-FEA)	11.86
		Butanol (W-FB)	4.12
		DI water (W-FW)	81.44
Crude ethanolic extract (E-C)	13.15	Hexane (E-FH)	3.73
		Ethyl acetate (E-FEA)	6.67
		Dichloromethane (E-FD)	15.47
		Ethanol (E-FE)	74.13

**Table 2:** Quantification of bioactive compounds of crude extracts and fractions of SJ by HPLC.

Bioactive Compounds	Concentration (mg/g Extract)				
	W-C	W-FW	E-C	E-FH	E-FE
Gallic acid	155.44 ± 4.90 ^c^	188.24 ± 2.15 ^b^	185.66 ± 5.71 ^b^	nd	204.91 ± 4.86 ^a^
Rutin	66.09 ± 2.47 ^c^	80.09 ± 3.94 ^b^	119.09 ± 4.65 ^a^	14.11 ± 2.22 ^d^	115.70 ± 3.16 ^a^
Chlorogenic acid	117.68 ± 3.56 ^c^	58.53 ± 3.53 ^d^	154.83 ± 3.82 ^b^	nd	169.55 ± 2.94 ^a^
Isoquercitrin	nd	nd	14.23 ± 1.74 ^a^	4.67 ± 0.77 ^b^	16.56 ± 1.37 ^a^
Quercetin	7.18 ± 1.14 ^b^	nd	25.44 ± 1.65 ^a^	5.46 ± 1.10 ^b^	27.81 ± 1.25 ^a^
Kaempferol	nd	nd	18.30 ± 1.69 ^b^	29.42 ± 2.18 ^a^	nd
Pyrogallol	30.69 ± 2.06 ^ab^	33.03 ± 1.68 ^a^	23.17 ± 1.83 ^c^	nd	27.17 ± 1.76 ^b^
Caffeic acid Apigenin	26.19 ± 1.15 ^b^ nd	18.64 ± 2.48 ^c^ nd	46.57 ± 2.20 ^a^ 21.30 ± 1.73 ^b^	nd 39.88 ± 1.64 ^a^	27.30 ± 1.25 ^b^ 2.75 ± 0.44 ^c^

All values are expressed as mean ± standard deviation (SD; *n* = 3). Different letters in each row indicate a significant difference (*p* < 0.05). nd = not detectable.

## Data Availability

The original contributions generated for this study are included in the article; the data presented in this study are available upon request from the corresponding author.

## Supplementary Materials

The following supporting information can be downloaded at: https://www.mdpi.com/article/10.3390/antiox14030252/s1. Figure S1: Steps of Research Methodology; Figure S2: Cell viability of MC3T3-E1 cells by PrestoBlue assay; Figure S3: HPLC chromatogram of mixed standards and E-FE fraction; and mass spectrum of major constituents; Table S1: List of chemicals and biological reagents and their manufacturer.
