# Supplementary material for: Osteoprotective Activity of Sambucus javanica Reinw Ex Blume subsp. javanica Leaf Extracts by Suppressing ROS Production
Source: Antioxidants (Basel). 2025 Feb 21;14(3):252. doi: 10.3390/antiox14030252 (PMC11939775; doi:10.3390/antiox14030252)
Supplement: Supplementary file 1 [file antioxidants-14-00252-s001.zip › antioxidants-3489825-supplementary.pdf]

Article

Osteoprotective Activity of *Sambucus javanica* Reinw Ex Blume Subsp. *javanica* Leaf Extracts by Suppressing ROS Production

Treethip Sukkho [1,2], Chartchai Khanongnuch [2,3], Saisamorn Lumyong [4,5], Jetsada Ruangsuriya [2,6], Sutasinee Apichai [2,7], Young-Joon Surh [8], Thanawat Pattananandecha [2,7*] and Chalermpong Saenjum [2,3,9*]

1 Department of Biotechnology School of Multidisciplinary and Interdisciplinary School Chiang Mai University, Chiang Mai 50200, Thailand; treethip.sk@gmail.com (T.S.)
2 Research Center for Innovation in Analytical Science and Technology for Biodiversity-Based Economic and Society (I-ANALY-S-T_B.BES-CMU), Multidisciplinary Research Institute (MDRI), Chiang Mai University, Chiang Mai 50200, Thailand; ck_biot@yahoo.com (C.K.); jetsada.ruang@cmu.ac.th (J.R.); thanawat.pdecha@gmail.com (T.P.); sutasinee.apichai@gmail.com (S.A.); chalermpong.saenjum@gmail.com (C.S.)
3 Research Center for Multidisciplinary Approaches to Miang, Multidisciplinary Research Institute (MDRI), Chiang Mai University, Chiang Mai 50200, Thailand.
4 Department of Biology Faculty of Sciences, Chiang Mai University, Chiang Mai 50200, Thailand; scboi009@gmail.com (S.L.)
5 Research Center of Microbial Diversity and Sustainable Utilization, Faculty of Science, Chaing Mai University, Chiang Mai 50200, Thailand.
6 Department of Biochemistry, Faculty of Medicine, Chiang Mai University, Chiang Mai 50200, Thailand.
7 Office of Research Administration, Chiang Mai University, Chiang Mai 50200, Thailand.
8 College of Pharmacy, Seoul National University, Seoul 08828, Republic of Korea; surh@snu.ac.kr (Y.J.S.)
9 Department of Pharmaceutical Sciences, Faculty of Pharmacy, Chiang Mai University, Chiang Mai 50200, Thailand.
* Correspondence: chalermpong.s@cmu.ac.th (C.S.); Tel.: +66-89-950-4227 (C.S.), thanawat.patt@cmu.ac.th (T.P.); Tel.: +66-93-146-3249 (T.P.)

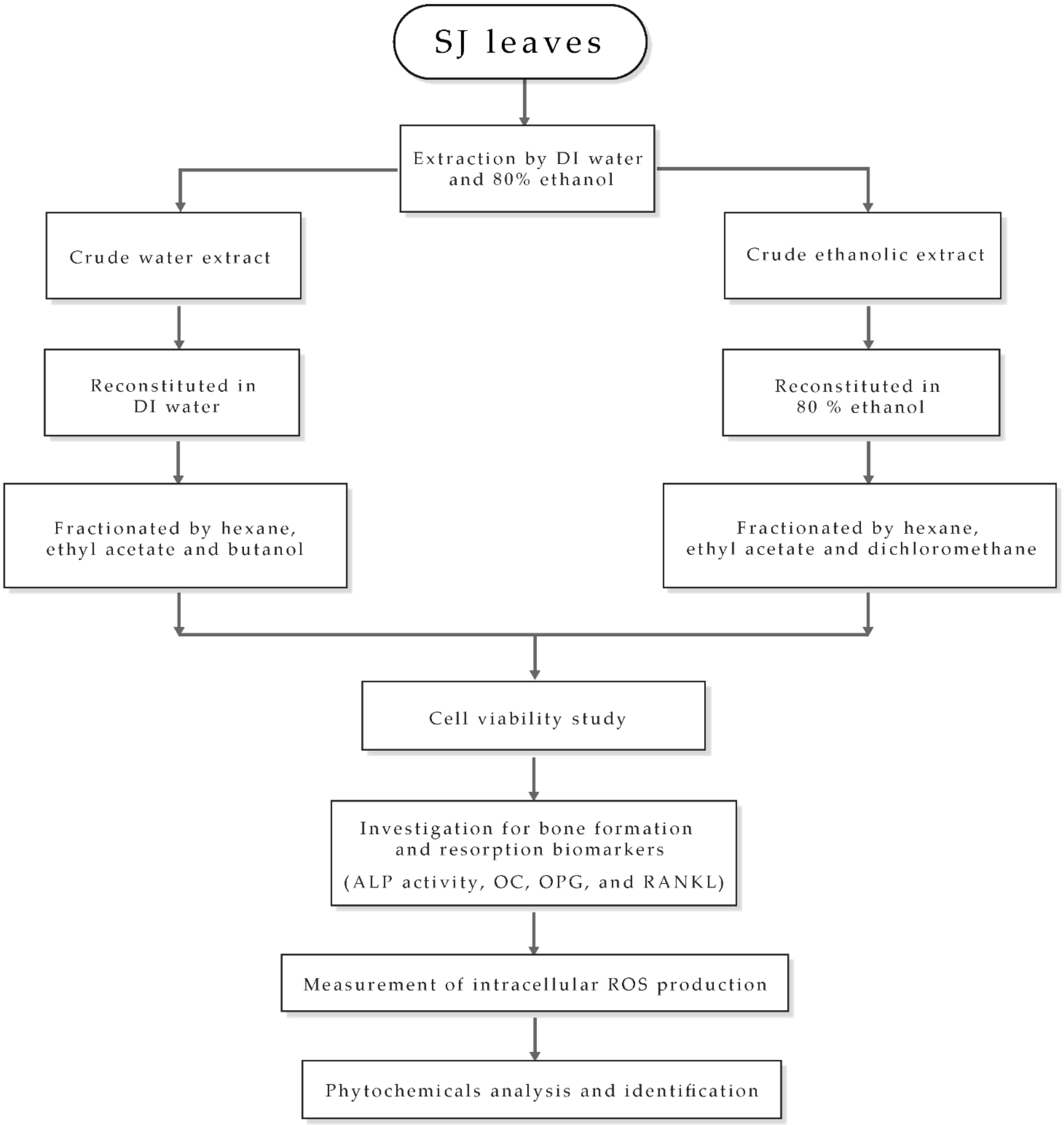

Figure S1. Steps of esearch methodology.

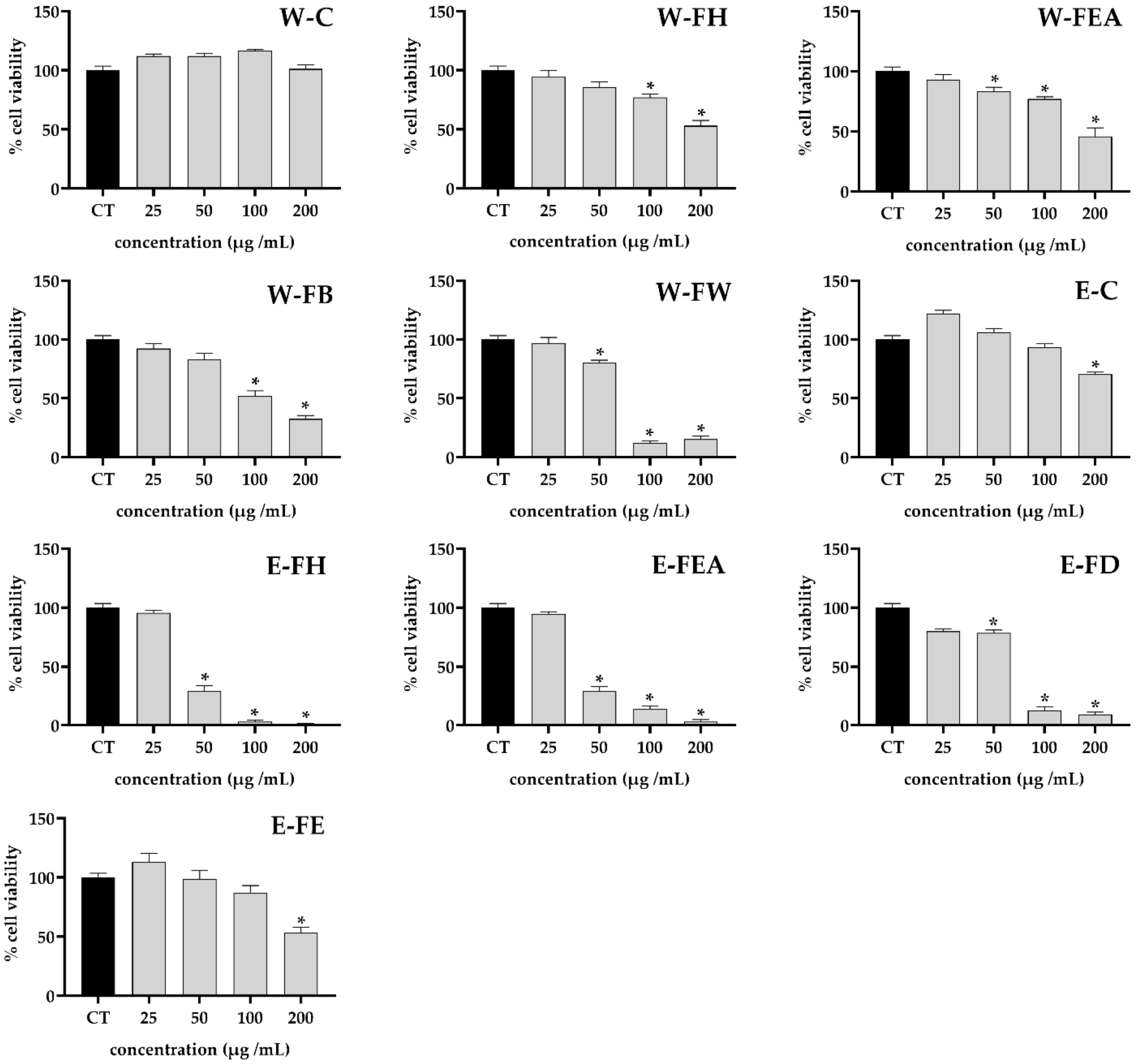

Figure S2. Cell viability of MC3T3-E1 cells by PrestoBlue assay. The cells were incubated in α MEM medium (control) or in α MEM medium supplemented with test samples at concentration ranges of 25 - 200 μg/mL of SJ extracts or fractions for 72 hours. * indicates a significant difference when compared to the control at P < 0.05.

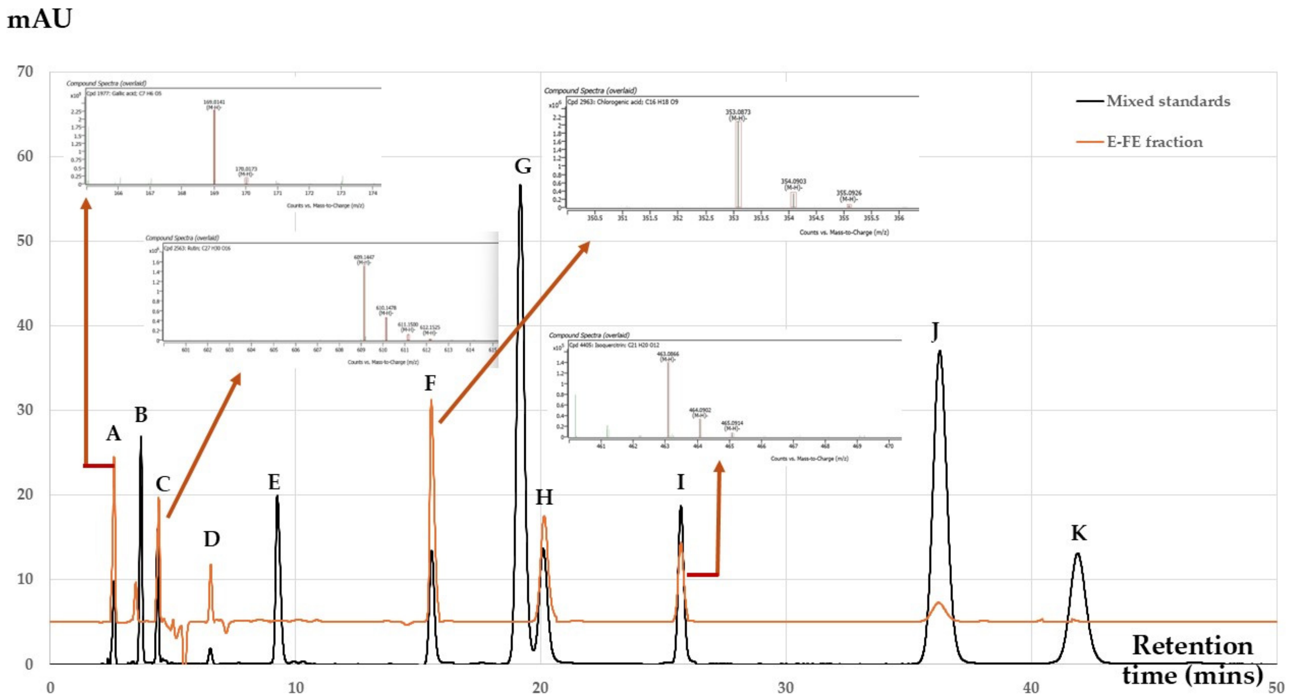

Figure S3. HPLC chromatogram of mixed standards and E-FE fraction; and mass spectrum of major constituents which (A) gallic acid, (B) caffeic acid, (C) rutin, (D) pyrogallol, (E) rosmarinic acid, (F) chlorogenic acid, (G) luteolin, (H) quercetin, (I) isoquercitrin, (J) Apigenin, and (K) kaempferol.

Table S1. List of chemicals and biological reagents and their manufacturer

Chemicals and Biological Reagents	Manufacturer‡
Alpha Minimum Essential Medium (αMEM), Fetal bovine serum (FBS), 0.5% trypsin-EDTA solution, Penicillin-streptomycin solution, Phosphate-buffered saline (pH 7.4)	Life Technologies (Paisley, UK)
PrestoBlue™ cell viability reagent	Life Technologies Corporation (Eugene, OR, USA)
Hydrochloric acid, Aluminum chloride ($AlCl_3$), Sodium hydroxide	Merck (Darmstadt, Germany)
Dimethylsulphoxide, Ethanol	RCI Labscan Limited (Bangkok, Thailand)
4-Nitrophenol magnesium chloride hexahydrate, Bovine serum albumin (BSA), CelLytic™ M cell lysis reagent, Diethanolamine, Bradford reagent, L-ascorbic acid (AsA), β-Glycerophosphate disodium salt hydrate (β-GP), Dexamethasone (DEX), Alizarin Red S, Ammonium hydroxide solution, Gallic acid monohydrate, 2′,7′-dichlorodihydrofluorescein diacetate (DCFH-DA), 4-nitrophenyl phosphate disodium salt hexahydrate	Sigma-Aldrich (St. Louis, MO, USA)
Chlorogenic acid	HWI group (Ruelzheim, Germany)
Rutin hydrate, Quercetin hydrate, Naringenin hydrate, Hesperidin, Kaempferol hydrate, Caffeic acid, Pyrogallol, Isoquercitrin	Tokyo Chemical Industry (Tokyo, Japan)
